# 

**Published:** 1895-11-30

**Authors:** 


### EARLY EDITION.

**Notices of Births, Deaths, and Marriages** are inserted in this column at a charge of 2s. 6d. for an announcement not exceeding four lines.

**Notice to Advertisers and Subscribers.**

All Advertisements, Orders for Copies of The Hospital and Business Communications should be addressed to **THE MANAGER, (Not to The Editor)**, The Hospital, 428, Strand, London, W.C.

The Cost of Subscription, post free, is as follows:—Per week, 2½d.; per quarter, 2s. 8d.; per half-year, 5s. 3d.; per annum, 10s. 6d. Foreign:—Per Annum, 15s. 2d.; payable, in all cases, in advance.

### NOTICE TO CORRESPONDENTS.

All MS., Letters, Books for Review, and other matters intended for the Editor should be addressed The Editor, The Lodge, Porchester Square, London, W.

The Editor cannot undertake to return rejected MS., even when accompanied by stamped directed envelope.

**"Burdett's Hospital Annual," 1895.**

Sixth year of publication. 950 pp. Scarlet cloth, gilt lettered. Price 5s. A most complete guide to British, Colonial, Indian, and American Hospitals, Dispensaries, Nursing and Convalescent Institutions, and Asylums. A few complete sets of the preceding five issues of the "Annual" may still be obtained on application to the Publishers, The Scientific Press (Limited), 428, Strand, London, W.C.

**NOTICE.—Vol. XVIII. of "The Hospital" (April to September, 1895), in handsome cloth binding, is now on sale at the Office of this Journal. Price 7s. 6d. Cases for binding the Numbers contained in this Volume, 1s. 6d. Index to Vol. XVIII., 2½d., post free.**

**The Monthly Part for December will be ready on December 1st, price 6d., by post, 8d.**

### Scraps and Gleanings.

The Hospital Sunday collections at Bootle amounted to the large sum of £419 13s. this year.

The Chelsea Hospital for Women has received a donation of £33 from the Hon. Mrs. L. Tollemache.

The Fishmongers' Company have granted 50 guineas to the Royal Westminster Ophthalmic Hospital.

An increase of nearly £500 marks the working men's collection to the Royal Infirmary at Newcastle-on-Tyne.

Extensive alterations and improvements are being effected in the operating theatre of the Cancer Hospital.

There were over 1,000 patients at the Royal Westminster Ophthalmic Hospital, King William Street, during last month.

A thousand pounds has been handed over to the secretary of the Coventry and Warwickshire Hospital from the Hospital Saturday collection.

The Duke of Westminster has forwarded a cheque of £600 for the Chester Infirmary, the amount of fees received from visitors to Eaton Hall.

A recreation ground for the patients is now being laid out at the City of Dublin Hospital, and a special fund for this purpose is being raised.

At the vegetarian congress which commenced at Birmingham last week, papers were read by delegates from America, Germany, Holland, India, &c.

Out of the annual cost of the Dublin Hospitals, which is about £49,000 a year, no less than 8 per cent. is supplied from the Hospital Sunday Fund.

The treasurer of Guy's Hospital has received a cheque of £1,000 from Mr. A. L. Cohen for the endowment of a bed in memory of his late son, Mr. H. A. Cohen.

A bazaar, in behalf of the Mary Wardell Convalescent Home for Scarlet Fever, will be held in the Iron Room, Stanmore Common, on Thursday, December 5th.

We regret to announce the death of Mr. Egerton Burnett, whose name is familiar to many of our readers who have utilised the excellent products of the firm he founded.

In connection with the financial position of the Royal Infirmary, Aberdeen, a house-to-house collection is being instituted in the surrounding villages on behalf of the hospital.

A munificent donation of £1,000 has been received by the managing committee of the Essex Convalescent Home at Clacton-on-Sea from Mr. H. C. Wells, of Bromfield Lodge.

The London Temperance Hospital has received £200 from the solicitors of the late Miss Houlton, also £100 from the executors of the late Mrs. Mary West, in discharge of legacies.

Paris has granted, through the municipal funds, a sum of money for new buildings to be constructed at the Salpêtrière Hospital for the treatment of nervous and mental affections.

The Metropolitan Hospital, Kingsland Road, has received a cheque for £140 as the result of the concert recently given at the Highbury Athenæum per Mr. P. W. Killby, hon. secretary.

### THE STUDENT OF MEDICINE.

### APPOINTMENTS.

P. S. Abraham, M.A., M.D., B.Sc., F.R.C.S.I., appointed Assistant Surgeon to the Hospital for Diseases of the Skin, Blackfriars. F. E. Adams, M.D.R.U.I., D.P.H.Camb., appointed Medical Officer of Health to the Hereford County Council. G. W. Barber, M.R.C.S.Eng., L.R.C.P.Lond., appointed Medical Officer to the Kalgoorlie (Hannan's) Hospital, West Australia. A. E. Broster, L.R.C.P.Edin., M.R.C.S.Eng., appointed Medical Officer to the Small-pox Hospital of the Wirksworth District Council. F. H. Carlyon, M.B., C.M.Edin., appointed Medical Officer to the Royal Cornwall Infirmary, Truro. T. H. M. Clarke, B.A., M.B., B.Ch., B.A.O.Dub.Univ., appointed Senior Resident Surgeon to the Jervis Street Hospital, Dublin. A. B. Davies, M.R.C.S.Eng., L.R.C.P.Lond., appointed Honorary Surgeon to the Stroud General Hospital. R. B. Duncan, M.D., B.S.B.Hy.Dunelm, appointed Medical Officer for the Bradninch District of the Tiverton Union. E. W. Hardwicke, B.A.Camb., M.B., B.C., appointed Medical Officer of Health to the Quarry Bank Urban District Council. C. M. Hardy, M.B.Durh., appointed Medical Officer of Health for the Darlington Rural Sanitary District. H. Harris, L.R.C.P.Edin., M.R.C.S.Eng., appointed Medical Officer for the Bacup District of the Haslingden Union. E. G. G. Little, M.D.Lond., B.A. CapeUniv., M.R.C.P.Lond., M.R.C.S Eng., appointed Assistant Physician to the East London Hospital for Children, Shadwell. F. W. D. McGachen, L.F.P.S.Glasg., L.S.A., D.P.H. Eng., appointed Medical Officer for the Markyate District of the Luton Union. O. J. Mackintosh, M.B., C.M.Edin., appointed Medical Officer and Public Vaccinator for the Parish of Portpatrick. W. F. Mellersh, L.D.S.R.C.S.Eng., appointed Honorary Dental Surgeon to the Thames Ditton Cottage Hospital. Mr. T. G. Matthews, appointed Medical Officer for the Sixth District of the Mansfield Union. T. Moore, M.R.C.S.Eng., L.S.A., appointed Medical Officer for the Hazelgrove, &c., District of the Stockport Rural District Council. E. S. Nutting, M.B., C.M.Edin., appointed Medical Officer of Health to the Warsop Urban District Council. H. D. Packer, M.R.C.S.Eng., L.R.C.P. Lond., appointed Assistant Resident Medical Officer to the East Riding Lunatic Asylum, Beverley, Yorks. J. Rowan, M.B., C.M., appointed Assistant Ophthalmic Surgeon to the Glasgow Ophthalmic Institution, Glasgow Royal Infirmary. E. Sharp, M.R.C.S.Eng., L.S.A., appointed Honorary Consulting Medical Officer to the Royal Cornwall Infirmary, Truro. H. C. Sharp, B.A., M.B.Camb., appointed Honorary Medical Officer to the Royal Cornwall Infirmary, Truro. E. Spencer, M.B., Ch.B.Vict., L.S.A., appointed Resident Medical Officer to the Colonial Hospital, Perth, Western Australia. Mr. D. Thomas, appointed Medical Officer of Health to the Ystradgynlais Rural District Council. E. M. Thuresson, L.R.C.P., L.R.C.S.Edin., appointed Medical Officer for the Second District of the Parish of St. George-in-the-East. A. McI. Tindall, M.R.C.S.Eng., L.S.A., appointed Medical Officer for the No. 2 District of the Market Harborough Union. A. Tuxford, M.D., appointed Medical Officer of Health to the Boston Town Council, the Port Sanitary Authority, Boston, and the Sebley Rural District. J. McD. Troup, M.A., L.S.A., appointed House Surgeon to King's College Hospital. P. C. E. Tribe, M.R.C.S., L.R.C.P., appointed House Surgeon to King's College Hospital. G. H. Lansdown, M.R.C.S., L.R.C.P., appointed Assistant House Physician to King's College Hospital. E. Playfair, M.B.Lond., M.R C.S., L.R.C.P., appointed Assistant House Accoucheur to King's College Hospital. J. K. Watson, M.B., C.M.Edin., appointed House Surgeon to the Essex and Colchester Hospital. H. A. Burridge, M.R.C.S., L.R.C.P., appointed House Accoucheur to King's College Hospital. J. S. Boden, M.R.C.S., L.R.C.P., appointed House Surgeon to King's College Hospital.

St. Thomas's Hospital House Appointments.—The following gentlemen have been selected as House Officers from Tuesday, December 3rd, 1895:—Resident House Physicians: F. B. Thornton, L.R.C.P., M.R.C.S., and W. E. Dixon, B.Sc.Lond., L.R.C.P., M.R.C.S. Non-Resident House Physicians: P. J. A. Seccombe, M.A.Cantab., L.R.C.P., M.R.C.S. (extension); and F. G. Layton, L.R.C.P., M.R.C.S. (extension). House Surgeons: E. O. Thurston, L.R.C.P., M.R.C.S. (extension); A. L. Home, L.R.C.P., M.R.C.S. (extension); W. G. Stone, M.A., M.B., B.Ch.Oxon., L.R.C.P., M.R,C.S. (extension); and H. J. Davis, M.A., M.B., B.C.Cantab., L.R.C.P., M.R.C.S. (extension). Assistant House Surgeons: L. A. R. Wallace, B.A., M.B., B.Ch.Oxon, L.R.C.P., M.R.C.S. (extension); H C. Crouch, L.R.C.P., M.R.C.S. (extension); J. L. Prain, L.R.C·P., M.R.C.S. (extension); and G. J. Conford, B.A., M·B., B.Ch.Oxon., L.R.C.P., M.R.C.S. (extension). Obstetric House Physicians: Senior, E. A. Saunders, M.A., M.B., B.Ch.Oxon., L.R.C.P., M.R.C.S.; and Junior, G. G. Genge, L.R.C.P., M.R.C.S. Ophthalmic House Surgeons: Senior, H. G. Toombs, L.R.C.P., M.R.C. (extension); and Junior, A. H. P. Dawnay, L.R.C.P., M.R.C.S. Clinical Assistants in the Special Department for Diseases of the Throat: J. W. Cornwall, M.A., M.B., B.C.Cantab. (extension); and W. Thornely, B.A.Cantab., L.R.C.P., M.R.C.S. Clinical Assistant in the Special Department for Diseases of the Skin: W. D. Knocker, L.R.C.P., M.R.C.S. Clinical Assistants in the Special Department for Diseases of the Ear: B. Dyball, L.R.C.P., M.R.C.S.; and P. W. Kent, L.R.C.P, M.R.C.S. Clinical Assistants in the Electrical Department: F. J. Brackenridge, L.R.C.P., M.R.C.S.; and W. D. Frazer, L.R.C.P., M.R.C.S.

### VACANCIES.

#### Resident Medical Officers.

Central London Ophthalmic Hospital, 238a, Gray's Inn Road, W.C.—House Surgeon. Rooms coals, and light. Applications to the Secretary by December 10th.

City of London Hospital for Diseases of the Chest, Victoria Park, E.—House Physician. Appointment for six months. Board and residence provided, and salary at the rate of £30 per annum. Applications to the Secretary by December 12th.

THE STUDENT OF MEDICINE—*continued.*

Denbighshire Infirmary, Denbigh.—House Surgeon; must be duly qualified to practise medicine and surgery, and be conversant with the Welsh language. Salary £80 per annum, with board, residence, and washing. Applications and testimonials to W. Vaughan Jones, Secretary, by December 2nd.

Ennis District Lunatic Asylum.—Assistant Medical Officer, doubly qualified, unmarried, and not more than 30 years of age. Salary £100 per annum, with furnished apartments, rations, washing, fuel, light, and attendance. Applications to Dr. Gelston, Resident Medical Superintendent, by December 13th.

General Hospital, Birmingham.—Assistant House Surgeon. Appointment for six months. No salary, but residence, board, and washing provided. Applications to Howard J. Collins, House Governor, by November 30th.

General Infirmary at Gloucester and the Gloucestershire Eye Institution. —House Surgeon; doubly qualified. Salary £100 per annum, with board, residence, and washing. The Assistant House Surgeon is a candidate for the post, and, if elected, the office of Assistant House Surgeon will be vacant. Candidates must be doubly qualified. Board, residence, and washing provided. Applications to the Secretary by November 30th.

Hospital for Diseases of the Throat, Golden Square, W.—Resident Medical Officer. Salary £50 per annum, with board, lodging, and washing. Applications to W. Holt, Secretary-Superintendent, by November 30th.

London Temperance Hospital, Hampstead Road, N.W.—Assistant Resident Medical Officer; doubly qualified. No salary, but board, washing, and residence provided. Applications to A. W. Bodger, Secretary, by December 6th.

Manchester Royal Infirmary.—Assistant Medical Officer at the Monsall Fever Hospital; unmarried. Appointment for twelve months. Salary £100 per annum, with board and residence. Applications to the Chairman of the Board, Royal Infirmary, Manchester, by November 30th.

North-Eastern Hospital for Children, Hackney Road, Shoreditch, N.E.—House Physician; doubly qualified. Appointment for six months; at the expiration of this term he will be required, if eligible, to serve as House Surgeon for a further period of six months. Salary as House Physician at the rate of £60, and as House Surgeon at the rate of £80 per annum. Junior House Physician for six months; doubly qualified. No salary, but board and lodging, including washing, provided. Applications to the Secretary, 27, Clement's Lane, E.C., by December 9th.

Royal Berks Hospital, Reading.—House Surgeon and House Physician. Salary in each case £60 per annum, with board, lodging, and washing. Also Assistant Medical Officer, with board, lodging, and washing provided, but no salary. Appointments for six months. Applications to the Secretary before December 9th.

St. George's and St. James's Dispensary, 60, King Street, Regent Street, W.—Resident Medical Officer. Salary £100 per annum, with furnished apartments, coals, and lighting; also Physician. Applications to the Secretary, St. Leger Bunnett, by December 4th.

Swansea General Hospital.—House Surgeon. Salary £50 per annum, with board, residence, washing, and attendance. Applications to Jno. W. Morris, Secretary, 9, Castle Street, Swansea, by December 16th.

Warneford Hospital, Leamington.—House Surgeon. Salary £100, with board, lodging, and washing. Appointment for six months, subject to re-election. Applications to the Secretary before December 14th.

**Non-Resident Medical Officers.**

City of Liverpool.—Assistant to the Medical Officer of Health, fully qualified, graduate of a British University, and possess diploma in Sanitary Science or Public Health. Salary £350 per annum, subject to a contribution of £3 per cent. to the Corporation Superannuation Fund. Not more than 35 years of age. Applications endorsed "Assistant the Medical Officer of Health" to be posted to the Town Clerk, Municipal Offices, Liverpool, to be delivered by November 30th.

County Borough of Bolton.—Medical Officer and Public Analyst; doubly qualified. Salary £400 per annum. Applications to R. G. Hinnell, Town Clerk, Town Hall, Bolton, by December 4th.

Hospital for Sick Children, Great Ormond Street, Bloomsbury.—House Surgeon to out-patients; non-resident. Appointment for six months but eligible for a second term. Salary 25 guineas. Applications to the Secretary, by December 3rd.

King's College, London.—Sambrook Surgical Registrarship. Applications (from King's College students only) to Walter Smith, Secretary by November 30th.

London Hospital, Whitechapel, E.—Dental Surgeon. Applications to the Governor by November 30th.

Royal Westminster Ophthalmic Hospital, King William Street, West Strand, W.C.—Clinical Assistants. Applications to T. Beattie-Campbell, Secretary, by November 30th.

Victoria University, Manchester.—External Examiner in Pharmacology and Therapeutics and in Surgery. Appointment for three years. Applications to Alfred T. Bentley, Registrar, by November 30th.

### Medical and Administrative Appointments.

**NORTH-EASTERN HOSPITAL FOR CHILDREN, HACKNEY ROAD, SHOREDITCH, N.E.**

HOUSE PHYSICIAN REQUIRED on January 1st. The appointment is made for six months, and, at the expiration of this term, the House Physician will be required, if eligible, to serve as House Surgeon for a further period of six months.

Salary as House Physician at the rate of £60 per annum, and as House Surgeon (the Senior post) at the rate of £80 per annum.

Candidates, who must possess a medical and surgical qualification, must send in their applications, with copies of not more than four testimonials, to the SECRETARY, at the City office, on or before Twelve noon on MONDAY, December 9th.

City Office, T. GLENTON-KERR, Secretary.
27, Clement's Lane, Lombard Street, E.C.,
November 13th, 1895. (1,102)

**BIRMINGHAM AND MIDLAND EYE HOSPITAL.**

WANTED, an ASSISTANT DISPENSER to attend at the above Hospital every Week-day Morning from Nine till One o'clock.

Applications addressed to the CHAIRMAN of the MEDICAL BOARD, stating age, qualifications, and salary required, must be sent on or before THURSDAY, the 12th day of December next.

Any further particulars may be obtained by applying to the SECRETARY.

By order,
CRANMER GELL, Secretary.

Secretary's Office,

Church Street, Birmingham,

November 25th, 1895. (1,371)

**NORTH-EASTERN HOSPITAL FOR CHILDREN, HACKNEY ROAD, SHOREDITCH, N.E.**

JUNIOR HOUSE PHYSICIAN REQUIRED for six months, commencing on January 1st.

Candidates must possess a medical and surgical qualification. No salary, but board and lodging (including washing) will be provided.

Applications, together with copies of not more than four testimonials, must be sent to the SECRETARY, at 27, Clement's Lane, E.C., not later than MONDAY, December 9th.

T. GLENTON-KERR, Secretary.
November 13th, 1895. (1,101)

**NORTH-EASTERN HOSPITAL FOR CHILDREN, HACKNEY ROAD, SHOREDITCH.**

An OPHTHALMIC SURGEON is REQUIRED (duties commencing from 1st January).

Candidates, who must possess a recognised British qualification in Surgery, should send in their applications, accompanied by copies of not more than four testimonials, on or before MONDAY, 9th December, to the Secretary at the City Office. T. GLENTON-KERR, Secretary.
City Office, 27, Clement's Lane, E.C. (1,266)

**YORK LUNATIC ASYLUM.**

WANTED, an ASSISTANT RESIDENT MEDICAL OFFICER. He must be duly qualified to act as a Surgeon, and will be required to devote the whole of his time to the discharge of his duties.

Salary £100 a year, with Board, Washing, and Attendance.

Applications, with testimonials, addressed to the COMMITTEE (under cover to me), must be sent in on or before Ten o'clock on WEDNESDAY MORNING, December 11th, 1895. R. D. HORNE, Secretary.
Bootham, York, November 26th, 1895. (1,325)

### FANNIN & COMPANY, DUBLIN.

### PUBLICATIONS.

**A MANUAL FOR MIDWIVES.** By Dr. FLEETWOOD CHURCHILL, Edited by THOS. MORE MADDEN, M.D., F.R.C.S.Ed., Obstetric Physician to Mater Misercordiæ Hospital. Late Master of the National Lying-in Hospital, Dublin.

Crown 8vo. 5th Edition. In the Press.

**CLINICAL SLATE.** Designed by HENRY DAVY, M.B., M.Ch., Univ. Dub., &c. For recording particulars of Private and Hospital Cases. 3s.

**A TEXT-BOOK OF CONTINUED AND ERUPTIVE FEVERS.**

By JOHN W. MOORE, B.A., M.D., M.Ch., Univ. Dub., F.R.C.P.I. With Charts and Illustrations. 15s.

**DUBLIN JOURNAL OF MEDICAL SCIENCE.**

Edited by JOHN W. MOORE, B.A., M.D., M.Ch., Univ. Dub. F.R.C.P.I., &c. Published monthly—containing Original Communications, Reviews, Abstracts, and Reports in Medicine, Surgery, and Collateral Science. Price 2s. each Number, or 20s. per annum.

### FANNIN AND COMPANY,

41, Grafton Street, Dublin,

MANUFACTURERS of SURGICAL INSTRUMENTS & APPLIANCES.

**Medical Booksellers and Publishers.**

Registered Telegraph Address—"Fannin, Dublin." Telephone No. 198.
Established upwards of Half a Century.

Medical Officer of Health.

By order,

M. M. BOWLAN, M.B.,

Medical Superintendent.

November 26th, 1895. (1,366)

### STANDARD MANUALS FOR NURSES.

Now ready, demy 8vo, blue buckram, gilt, 3s. 6d., with numerous illustrations.

**DIET IN SICKNESS AND IN HEALTH.** By Mrs. ERNEST HART, formerly Student of the Faculty of Medicine of Paris, and of the London School of Medicine for Women. With an introduction by Sir Henry Thompson, F.R.C.S., M.B. Lond.

"Mrs. Ernest Hart, in consequence of her scientific training, of her intimate association with things medical, and of her evident acquaintance with the practical details of cooking, is singularly well fitted to write upon the subject of diet. To nurses also the many recipes given, and the clear account of the principles which should underlie the arrangement of any invalid diet, must be of the utmost value."—*Daily Chronicle.*

THIRD AND REVISED EDITION. Demy 8vo, 209 pp., profusely Illustrated with Copyright Portraits and Drawings. Price 2s. 6d., post free.

**HOW TO BECOME A NURSE: AND HOW TO SUCCEED.** A complete guide to the nursing profession for those who wish to become Nurses, and a useful book of Reference for Nurses who have completed their training and seek employment. Compiled by HONNOR MORTEN. Author of "Sketches of Hospital Life," "The Nurse's Dictionary," &c.

"To those who are frequently appealed to by girls, or young women, as to the steps they should take to become nurses, this book of Miss Morten's must prove a perfect godsend."—*British Medical Journal.*

SECOND AND REVISED EDITION. Demy 16mo, suitable for the apron pocket, handsomely bound in cloth, 140 pp. Price 2s.; in handsome leather, gilt, price 2s. 6d. net.

**THE NURSES' DICTIONARY OF MEDICAL TERMS AND NURSING TREATMENT.** Compiled for the use of Nurses, by HONNOR MORTEN. Containing descriptions of the principal Medical and Nursing Terms and Abbreviations, Instruments, Drugs, Diseases, Accidents, Treatments, Physiological Names, Operations, Foods, Appliances, &c., &c., encountered in the Ward or Sick-room.

"Should be at the disposal of every nurse."—*Birmingham Medical Review.*

Third Edition. Enlarged and partly rewritten, crown 8vo, cloth gilt, about 200 pp., price 2s. 6d.

**HOSPITAL SISTERS AND THEIR DUTIES.** By EVA C. LUCKES, Matron to the London Hospital. The favourable reception accorded to the first two editions of this useful and interesting work, and the regular demand for copies of it, encourage the publishers to place this third edition, greatly improved and enlarged, before the Institutions. The experience and position of its author entitle it to authority, and every Matron, Sister, or Nurse who aspires to become a Sister should read the advice and information contained in its pages.

Important New Handbook of Midwifery. Handsomely bound in leather, 260 pp., profusely illustrated, price 6s., post free.

**A PRACTICAL HANDBOOK OF MIDWIFERY.** By FRANCIS W. N. HAULTAIN, M.D. A Practical Manual produced in a portable and convenient form for reference.

"The book is one of the best of its kind, and is well fitted to perform the functions its author claims for it."—*Edinburgh Medical Journal.*

Demy 8vo, nearly 200 pp., cloth gilt, profusely illustrated with over 70 special cuts. Price 6s. (post free).

**THE ART OF MASSAGE.** By A. CREIGHTON HALE. A complete Text-book of this valuable art. The most practical, simple, and generally useful work published on the subject. With fully illustrated chapters on the Anatomy of the Human Body.

London: THE SCIENTIFIC PRESS (Limited), 428, STRAND, W.C.

### "THE HOSPITAL"

AN INDEPENDENT JOURNAL OF

**The Medical Sciences**

AND

**Hospital Administration,**

WITH WHICH IS ISSUED WEEKLY

**A SPECIAL**

### SUPPLEMENT FOR NURSES.

PUBLISHED EVERY SATURDAY.

PRICE TWOPENCE.

Matrons, Sisters, or Nurses will find in THE HOSPITAL a chronicle of the latest Nursing News, Practical Articles of the highest value to those who wish to keep themselves abreast with the times, and brightly written articles from Nurses all over the world.

**SUBSCRIPTIONS CAN COMMENCE AT ANY TIME.**

### TERMS OF SUBSCRIPTION.

### WEEKLY ISSUE.

| | Great Britain. | Foreign and Colonial. |
|---|---|---|
| For One Year ... | 10s. 6d. ... | 15s. 2d. |
| For Six Months ... | 5s. 3d. ... | 7s. 7d. |
| For Three Months... | 2s. 8d. ... | 3s. 10d. |

### MONTHLY PARTS.

| | Post Free to any Part of the World. |
|---|---|
| For One Year ... | ... 8s. 6d. |
| For Six Months ... | ... 4s. 3d. |
| For Three Months ... | ... 2s. 2d. |

Postal Orders and Cheques should be made payable to "The Scientific Press, Limited."

N.B.—In order to prevent delay, all business communications should be addressed to "THE MANAGER" only, and not to any person individually.

### CHANGE OF ADDRESS.

In event of change of address, notice should be forwarded to the Publishers by the Saturday previous to date of publication, and the old address should also be given.

"THE HOSPITAL" can be obtained of all Booksellers and Newsagents in the United Kingdom, and at all the Railway Stalls, of Messrs. W. H. Smith and Son; and also from the following Agents in the Colonies and Abroad:—

PARIS: Librairie Galignani, 224, Rue de Rivoli.
BERLIN: Messrs. A. Asher and Co.
LEIPSIG: Mr. F. A. Brockhaus.
NEW YORK: The International News Co.
MONTREAL: The Montreal News Co.
TORONTO: The Toronto News Co.
MELBOURNE, SYDNEY, ADELAIDE, and NEW ZEALAND: Messrs. Gordon and Gotch.
INDIA: At all the Railway Bookstalls and Agencies of Messrs. A. H. Wheeler and Co.
SOUTH AFRICA: Messrs. Juta, Cape Town.

PUBLISHING OFFICES: 428, STRAND, LONDON, W.C.

### IMPORTANT NOTICE.

OUR ISSUE OF

**DECEMBER 14TH**

WILL CONSIST OF A

**SPECIAL DOUBLE**

**CHRISTMAS NUMBER,**

With which will be presented a handsome Presentation Plate of

**H.R.H. THE PRINCE OF WALES,**

Taken from an entirely new and hitherto unpublished photograph.

This plate will be a companion plate to that of H.R.H. the Princess of Wales, given with our Christmas Number in December last.

**2d. PRICE AS USUAL. 2d.**

In order to secure copies orders should be sent in at once to

THE MANAGER,

"THE HOSPITAL," 428, STRAND, LONDON, W.C.

